# A Southern Ocean trigger for Northwest Pacific ventilation during the Holocene?

**DOI:** 10.1038/srep04046

**Published:** 2014-02-17

**Authors:** S. F. Rella, M. Uchida

**Affiliations:** 1 National Institute for Environmental Studies (NIES), Center for Environmental Measurement and Analysis, Onogawa 16-2, Tsukuba 305-8506, Japan; 2 Japan Agency for Marine-Earth Science and Technology (JAMSTEC), the Research Institute for Global Change (RIGC), 15-2 Natsushima-cho, Yokosuka, 237-0061, Japan

## Abstract

Holocene ocean circulation is poorly understood due to sparsity of dateable marine archives with submillennial-scale resolution. Here we present a record of mid-depth water radiocarbon contents in the Northwest (NW) Pacific Ocean over the last 12.000 years, which shows remarkable millennial-scale variations relative to changes in atmospheric radiocarbon inventory. Apparent decoupling of these variations from regional ventilation and mixing processes leads us to the suggestion that the mid-depth NW Pacific may have responded to changes in Southern Ocean overturning forced by latitudinal displacements of the southern westerly winds. By inference, a tendency of in-phase related North Atlantic and Southern Ocean overturning would argue against the development of a steady bipolar seesaw regime during the Holocene.

The importance of global ocean circulation for climate variability is now well recognized^1^. During the last glacial and deglacial period, millennial-scale cold episodes in the high latitude Northern Hemisphere appear to have been associated with relatively warm episodes in the high latitude Southern Hemisphere, possibly mediated by flip-flops in North Atlantic and Southern Ocean deep water formation rates, a behaviour of the climate system termed the bipolar seesaw^2^. The role of Southern Ocean overturning has been recently also highlighted by a reconstruction of the relative strength of deep water flows from the Southern and North Atlantic oceans, which suggested a South-North flow during the Last Glacial Maximum^3^. By contrast, the climatic and oceanic history of the last ~11.6 kyr (kyr denotes a time span of 1000 years), the Holocene, has been long regarded as comparatively stable. However, recent studies demonstrated that an insolation-driven southward shift of the summer position of the Intertropical Convergence Zone over the course of the Holocene^4^ in association with progressive weakening of the monsoon systems in Africa and Asia^5^,^6^ and increasing El Niño Southern Oscillation variability^7^,^8^ resulted in considerable global and local changes in atmospheric circulation, precipitation patterns and sea surface temperatures (SST) that severely impacted human civilizations^9^. Remarkably, the role of the global ocean for these climatic changes is still poorly understood, despite its strong potential to modulate climate through heat transport and air-sea trace gas exchange^10^. In particular, the Holocene oceanic histories of the Southern and Pacific oceans are largely unknown, because sedimentation rates were typically low and calcium carbonate preservation poor precluding high resolution studies^11^.

In response to these challenges, a high sedimentation piston core (PC C9002A) was recovered in 2005 during cruise CK05-04 of R/V Chikyu from off Shimokita peninsula (41°11′01.20″N, 142°12′01.97″E) at 1179 m water depth^12^ (Fig. 1) (see Methods). Surface waters off Shimokita peninsula are today dominantly influenced by a branch of the Tsushima current, the Tsugaru current^13^, which flows from the Japan Sea through the Tsugaru Strait to the NW Pacific over a shallow sill of ~50 m depth, and to a lesser extent by the Oyashio current that flows southward along the Kuril Arc^14^ (Fig. 1).

The deep North Pacific Ocean contains the oldest waters of the global ocean, which originate almost entirely from the Southern Ocean and North Atlantic with unequal contributions of ~70% and ~25%, respectively^15^,^16^. Principal Southern Component Waters (SCW) reaching the North Pacific with a combined strength of 24 Sverdrup (Sv) are the Lower Circumpolar Deep Water (LCDW; ~4000 m depth) that includes a contribution of modified NADW, the Upper Circumpolar Deep Water (UCDW; ~3000 m depth) and the Antarctic Intermediate Water (AAIW; ~1000 m depth)^17^ (Fig. 2). LCDW is channelled northward through passages in the complex topography of the Western Pacific, flowing by the northeastern coast of Japan at a strength of 6 Sv. UCDW largely flows into the Philippine Sea and further on towards the Hawaiian islands. AAIW spreads from the Southeast Pacific northwestward to ca. 20°N^17^ (Fig. 2). Modern highly ^14^C-depleted waters with a radiocarbon content (Δ^14^C) of ~−195‰ at a water depth of ~1200 m off northeastern Japan^18^ demonstrate that the mid-depth waters off Shimokita peninsula are at present influenced by these old water masses and may so have been variably in the past.

Deep convection in the North Pacific, on the other hand, is very weak or absent today due to the low salinity lid of the North Pacific Ocean and water vapour transport related to the Asian Monsoon^19^. Evidence from radiolarian assemblages in the Bering Sea, a possible source of intermediate or deep water formation^20^, suggests that North Pacific ventilation was weak or absent all through the Holocene^21^,^22^. Nonetheless, shallow convection occurs today in the Okhotsk Sea due to winter time brine rejection^23^ leading to a characteristic low-salinity high-oxygen water mass between ~300 to ~800 m depths in the subtropical North Pacific, known as North Pacific Intermediate Water (NPIW)^24^. Considering the proximity of our study site to the Okhotsk Sea, episodes of presumably deeper reaching NPIW ventilation may have also affected upper mid-depth waters^25^.

In the present study we aim to reconstruct for the first time the Holocene ventilation history of Northwest (NW) Pacific mid-depth waters and discuss potential causes of its variability.

## Results

### Variations in mid-depth water Δ^14^C off Shimokita over the last 12 kyr

Holocene radiocarbon contents at site C9002A are presented in Fig. 3b and Table 1. Δ^14^C decrease from −37‰ at ~11.6 ka (ka denotes 1000 years before present) to values around −114‰ between ~9.2 and ~8.1 ka. Subsequently, values increase towards −66‰ at ~6.9 ka and are still elevated (−74‰) at ~6.2 ka. After ~6.2 ka, Δ^14^C decrease in two steps towards −252‰ at ~1.4 ka. A plateau with relatively constant Δ^14^C of ~−148‰ is evident between ~4.9 and ~3.1 ka. During the last ~1.4 kyr values increase to −200‰ at 0.5 ka, which is close to the modern value of ~−195‰^18^.

### Variations in mid-depth water projection ages off Shimokita over the last 12 kyr

Trends in radiocarbon propagate from the atmosphere to the ocean, resulting in a similar, but generally time-displaced trend of atmospheric and oceanic Δ^14^C at orbital time scales^26^,^27^ (Fig. 3a, b). Because ocean circulation changes occur at time scales of ~500 to ~2000 yr, the atmospheric and oceanic ^14^C trends can differ considerably at millennial scales, a practical phenomenon to deduce past ventilation states of water masses^16^,^28^. Methodologically, we apply the projection age method^28^, assuming a constant average reservoir age of 1000 yr in the two potentially dominant source regions, the Southern Ocean and the Okhotsk Sea (see Methods). Prior to ~8.1 ka, we additionally show projection ages under a scenario of an average source regions reservoir age of 1400 yr to take account of possibly highly increased reservoir ages of ~1900 yr in the Southern Ocean^29^ (see Methods).

Variations in projection ages over the last 12 kyr are presented in Figs. 3c, 5e, 6f and Table 1. Projection ages increase from ~820 yr at ~11.6 ka to average values of ~1180 yr between ~10.8 and ~9.1 ka. Subsequently, values decrease to ~900 yr between ~8.7 and ~8.1 ka. In the scenario with a source regions average reservoir age of 1400 yr, projection ages are 400 yr younger between 11.6 and 8.1 ka. At some time between ~8.1 and ~7.3 ka, projection ages strongly decrease to average values of ~270 yr between ~7.3 and ~6.2 ka. Thereafter, they increase in two steps towards ~1600 yr at ~1.4 ka. A plateau with relatively constant projection ages of ~880 yr is evident between ~5.4 and ~3.1 ka, whereby slightly lower values of ~800 yr occur between ~4.3 and ~3.6 ka. Between ~0.8 and ~0.4 ka values average ~930 yr. Transition times of major changes in projection ages are at the order of ~400 to ~800 yr.

## Discussion

Assessment of the potential influence of local and global water masses on projection ages at site C9002A throughout the Holocene requires knowledge of changes in regional ventilation and mixing processes, Southern Ocean overturning and NADW formation. While a few records are available for the North Atlantic^30^, dateable Holocene marine archives with sufficient time resolution are rare in the Southern and Pacific oceans^11^. One possibility to circumvent observational limits is to resort to the reconstruction of atmospheric and surface ocean conditions, which precondition ocean overturning and mixing.

Although mid-depth waters off Shimokita are today influenced by water masses from the North Atlantic and Southern Ocean, two major regional processes could have altered mid-depth ventilation conditions in the NW Pacific through time, variations in direct ventilation from the Okhotsk Sea and mixing processes at the confluence of the Kuroshio and Oyashio currents^31^ (Fig. 4). Direct ventilation of Okhotsk Sea Mode Water (OSMW), an important precursor for NPIW, is tightly coupled to winter-time northwesterly winds over the Okhotsk Sea, which push sea ice away from the coast and support brine rejection around polynyas and subsequent sinking of dense shelf waters^32^ (Fig. 4). Conditions of Arctic air surging into the mid-latitudes over the Okhotsk Sea is a typical expression of the negative mode of the Arctic Oscillation (AO)^33^,^34^, while the positive AO mode is associated with mean warmer conditions over Eurasia^34^,^35^. The positive AO mode is particularly effective in increasing Amur river discharge and autumn SST, thus decreasing winter sea ice in the Okhotsk Sea^35^. Evidence points to a positive mean state of the AO during the early and mid-Holocene changing to a negative mean state in the late Holocene^36^,^37^, which would be in line with climate evolution in eastern Siberia, the land mass adjacent to the Okhotsk Sea, characterized by a wet, albeit relatively cool early Holocene, a warmer mid-Holocene, and a cool late Holocene with increasing temperatures since ~3 ka^38^ (Figs. 4, 5a). Consistently, alkenone studies in the Okhotsk Sea suggest variable and moderate autumn SST in the early Holocene, relatively warm SST during the mid-Holocene and a tendency of decreasing SST thereafter^39^,^40^ with the exception of increasing tendencies since ~3 ka in the central part of the Okhotsk Sea^40^ (Figs. 4, 5b). Long-term changes in the AO mode and climate in Eastern Siberia and associated effects on SST and sea ice would thus call for moderate NPIW formation rates during the early Holocene, low rates during the mid-Holocene, increasing rates at the transition to the late Holocene and possibly somewhat decreasing rates since ~3 ka. Such inference is consistent with a recent high resolution record on carbon and oxygen isotopes of planktonic and benthic foraminifera combined with nutrient proxies between 600 and 1000 m depth in the Okhotsk Sea, suggesting that NPIW was weaker than today during the early and mid-Holocene and shifted since ~4.5 ka to a more modern-like state^41^. Consideration of atmospheric teleconnections leads to similar conclusions: General trends of decreasing SST in the North Atlantic Ocean during the Holocene tend to be in phase with Okhotsk Sea SST, while they tend to be out of phase with Bering Sea SST^39^, an observation reminiscent of a prominent role of the AO that tends to promote wind and temperature patterns of opposite signs in the Okhotsk and Bering seas^42^,^43^. Projection ages off Shimokita peninsula essentially indicate increasing ventilation between the early and mid-Holocene, followed by a stepwise decreasing trend until the late Holocene and rejuvenation since ~1.4 ka (Fig. 5e). These data thus largely decouple from surface water and climate conditions in the Okhotsk Sea area, challenging the idea of appreciable control of NPIW formation on NW Pacific mid-depth water ventilation during the Holocene.

Apart from direct ventilation in the Okhotsk Sea, mixing processes related to the confluence of the Oyashio and Kuroshio currents might lead to downward propagation of Δ^14^C signals from the surface ocean to site C9002A. Today, the main mixing zone occurs off northeastern Honshu Island affecting waters to a few 100 m depths, thereby essentially setting the properties of NPIW^44^. Today, these mixing processes considerably increase surface productivity, in particular biogenic opal, due to upwelling of nutrient-rich deeper waters giving rise to one of the most important fishing grounds^31^,^45^. Periods of intensified mixing would thus be expected to imprint on the sedimentary biogenic opal record. Yet, a Holocene biogenic opal record from 2215 m depth southeast of site C9002A indicates low biogenic opal contents between the late deglaciation and mid-Holocene with values increasing since ~5 ka^45^ (Fig. 5d). That mixing intensity is important in this region for changes in primary productivity rather than SST is apparent in continuously low biogenic opal contents between ~12 and ~5 ka, despite considerable changes in SST^45^ (Fig. 5c, d). According to these observations, deep mixing appears unlikely a primary cause for observed ventilation changes off Shimokita peninsula.

**Figure 2:**
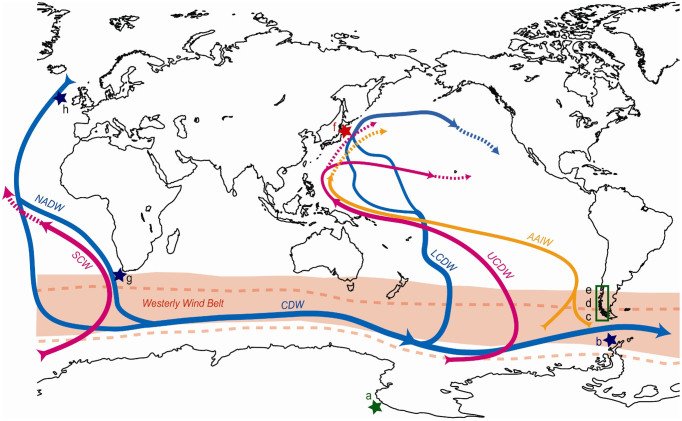
Illustration of deep and intermediate water mass distributions discussed in this study. Continuous arrows indicate modern and dashed arrows inferred mid-Holocene (~7 ka) conditions (CDW = Circumpolar Deep Water; LCDW = Lower Circumpolar Deep Water; UCDW = Upper Circumpolar Deep Water; AAIW = Antarctic Intermediate Water; SCW = Southern Component Water; NADW = North Atlantic Deep Water). Core locations discussed are indicated by solid star symbols and a green rectangle over the latitudinal range between ~40°S and ~55°S for the southern Chile westerly wind records. Single letters refer to corresponding panels in Fig. 6. Pink shading schematically denotes the extent of the southern westerly winds (SWW) in their northern position based on an analogy of northward shifted SWW to modern austral winter-like conditions^47^,^52^. Pink dashed demarcations indicate SWW in their southern position based on an analogy to modern austral summer-like conditions^47^,^52^. Blank world map source: http://english.freemap.jp/world_e/8.html/.

Above considerations on apparent inefficiency of regional processes to explain variations in projection ages motivate considerations of deeper water influencing the ventilation state of mid-depth waters. A first indication for such surmise illustrates a marked mid- to early late Holocene increase in the benthic species *Elphidium batialis* at ~1100 m depth east of Hokkaido (Fig. 4) that could be related to the influence of higher oxygen deeper waters, where *E. batialis* commonly dwells, invading the otherwise present oxygen minimum zone^21^. Considering the flow-by of Antarctic-derived LCDW east of Japan and AAIW and UCDW spreading towards the Northwest Pacific (Fig. 2), such deep-sourced ventilation of mid-depth waters could be related to changes in intermediate and deep water production in the Southern Ocean. In such configuration, instances of water mass rejuvenation at mid-depths in the NW Pacific may be envisaged as the advection of younger water masses in response to invigorated convective processes in the Southern Ocean, possibly complemented by mixing with younger overlying NPIW.

Discussion of southern-sourced changes in NW Pacific mid-depth waters requires a reconstruction of convective intensities in the Southern Ocean over the Holocene, for which we resort to the reconstruction of atmospheric and surface ocean conditions due to aforementioned sparsity in dateable marine archives in the Southern Ocean. Recent observation and model studies highlight the importance of variations in the latitudinal position, extent and intensity of the southern westerly winds (SWW)^46^,^47^ for providing the mixing needed to return deep waters to the surface of the Southern Ocean^48^,^49^,^50^. Mechanistically, polar easterly winds over Antarctica create the eastward flowing Antarctic Coastal Current and SWW the westward Antarctic Circumpolar Current (ACC) that promote divergence and upwelling of relatively salty Circumpolar Deep Water (CDW)^51^. Waters deflected towards the Antarctic increase in density due to cooling and brine rejection, consequently sink, mix with CDW and form Antarctic Bottom Waters. Waters deflected northwards sink at the Antarctic Frontal Zone below less dense subantarctic waters to form AAIW^51^. Mean poleward shifted and commonly intensified SWW, such as occur during austral summers today^47^,^52^ (Fig. 2), enhance overturning that in turn leads to a decrease of annual sea ice duration, surface waters warming and heat release to the atmosphere^51^,^53^,^54^.

Accordingly, in order to investigate possible climatic and oceanic links between the Holocene Southern and NW Pacific oceans, we compare the ventilation record from off Shimokita (Fig. 6f) to variations in Antarctic air temperature (AAT) based on Taylor Dome hydrogen isotopes^53^ (Fig. 6a), TEX_86_-based SST from off western Antarctic Peninsula (WAP)^49^ (Fig. 6b), the position and intensity of SWW^47^,^55^ (Fig. 6c–e), and intensities of SCW and NADW (Fig. 6g, h)^3^,^29^. Locations of proxy records are indicated in Fig. 2.

SST off WAP are particularly sensitive to upwelling of CDW through early spring sea ice retreat and the positions of ACC and SWW^49^,^56^. The clay/silt ratio from the Seno Skyring fjord system in southern Chile (~53°S) (Fig. 6c) can be interpreted in terms of a southward displacement of SWW leading to stronger winds over the fjord system that are reflected in increased clay advection from the Andes^47^. At the same latitude, biogenic carbonate accumulation rates (bio-CaCO_3_ AR) (Fig. 6d) in a fjord ~70 km east of the western entrance of the Magellan Strait trace the influence of open marine waters in the fjord due to their dependence on fjord surface water salinity levels, whereby low bio-CaCO_3_ AR and low salinities are associated with increased precipitation and southward-shifted, stronger SWW, which keep low salinity waters inside the fjord system^47^. The Fe XRF-scanner intensity data from the southern Chile continental slope (41°S)^55^ (Fig. 6e), a location sensitive to changes in the latitudinal position of SWW, depends primarily on the precipitation-driven relative contribution of iron-poor material from the low altitude coastal range, which is delivered to the slope by rivers. Accordingly, increases in Fe intensity trace more arid phases and by implication more poleward shifted SWW^55^.

At precessional scales, the AAT and WAP SST records show a decreasing trend over the course of the Holocene until ~2 ka (Fig. 6a, b), which is thought to be primarily driven by shortening of Antarctic summers, apparently in response to declining austral spring insolation at ~65°S (ref. 49,57). Over the same period, SWW shifted or expanded progressively to the north based on the southern Chile records (Fig. 6c–e). During the last ~2 kyr, on average, SST and AAT tend to increase and SWW to shift southward.

As illustrated in Fig. 6, AAT, WAP SST and SWW trends in the Antarctic sector are essentially mimicked by projection age changes off Shimokita that show a trend from high to low ventilation between ~7.3 and ~1.4 ka and a recovery in ventilation thereafter. Also the projection age plateau between ~5.4 and ~3.1 ka is reflected in similar plateaus in the AAT and SWW records and in a renewed SST increase off WAP. During the early Holocene, on the other hand, higher AAT, WAP SST and a more poleward position of SWW seem to facilitate enhanced Southern Ocean overturning, which is, however, not reflected in the NW Pacific mid-depth waters (Fig. 6) when assuming a source regions reservoir age of 1000 yr. Possibly subdued overturning in the Southern Ocean could be related to fresh water impacts from the melting Antarctic ice sheet^58^,^59^. Van Beek et al.^28^, on the other hand, observed exceptionally old Southern Ocean reservoir ages of ~1900 yr during the early Holocene, which would lead to projection ages off Shimokita intermediate between the mid- and early late Holocene (Fig. 6f; see Methods) and thus support an early Holocene link between Southern Hemisphere (SH) records and NW Pacific mid-depth ventilation.

In order to investigate causal relationships between SH records and projection ages (assuming a Southern Ocean reservoir age of ~1900 yr prior to 8.1 ka) in more detail, cross-correlations were examined for time lags, where projection ages lag SH proxies by ~500 ± 300 yr, the time scale of water flow between the Southern Ocean and the North Pacific^16^,^60^ with deviations that may have occurred during the Holocene. A summary and illustrations of cross-correlations are presented in Supplementary Material. SH proxies and projection ages are significantly correlated (n = 50, p < 0.01) within millennial-scale time windows around the ~500 yr lag, which highlights, in particular, their similarities in trends. In this context, the general AAT and SST cooling and SWW northward shift between the early and late Holocene could be related to the trend in ventilation off Shimokita. When emphasizing trend-corrected variabilities by correlating the first differences of SH proxies and projection ages, correlations show peaks of significant values within lags of ~500 ± 300 yr for SST (n = 50; p < 0.01) for the periods after 12 ka and after 8.1 ka, and for bio-CaCO_3_ AR (n = 50; p < 0.05) after 8.1 ka. The period after 8.1 ka excludes the early Holocene, where processes linking AAT, SST and SSW to ocean overturning could have been biased. First difference correlations would be also significant (n = 50; p < 0.05) after 8.1 ka for AAT and the clay/silt ratio within the ~500 ± 300 yr lag window, when taking into account combined uncertainties in age models at the order of respectively ±~500 yr^53^,^61^ and ±~700 yr estimated based on uncertainties and the resolution of age controls and the variability of sedimentation rates^47^. In case of the Fe intensity record, first difference correlations are not significant (n = 50, p < 0.05), which could be related to centennial- to millennial-scale deviations from the premise that wetness changes trace SWW changes^55^.

The temporal variations of SH records compared to projection ages thus substantiates the idea that projection ages could be causally related to formation and spreading of LCDW, UCDW and/or AAIW, at least for the period after 8.1 ka (Fig. 2). In this context, the ~400 to ~800 yr transition times between major ventilation changes off Shimokita may reflect waxing and waning of transpacific water transport.

To further examine the possibility of changing Southern Ocean overturning during the Holocene from an oceanic perspective, we compared our NW Pacific ventilation record with a mid-deep water ^231^Pa/^230^Th record (core MD02-2594; 2440 m water depth) from Cape Basin off South Africa in the Southeast Atlantic Ocean, ideally located to trace the respective importance of northern and southern component waters^3^ (Figs. 2, 6g). A temporary decrease in the ^231^Pa/^230^Th ratio indicates reduced NADW and/or enhanced SCW intensities^3^. Thus, NADW apparently increased and/or SCW decreased between ~12 and ~2.8 ka, while the opposite situation occurred thereafter. At millennial scales, the ^231^Pa/^230^Th record plateaus between ~7.5 and ~6 ka and shows temporarily decreased values at ~3.9 ka, which could be interpreted in terms of stabilization or reduction of NADW and/or enhancement of SCW. Constraints to the relative importance of northern and southern deep water mass fluxes provides a record of NADW variability based on changes in δ^13^C of the benthic foraminifera *Cibicidoides Wuellerstorfi* at 2179 m water depth in the eastern North Atlantic^29^ (Figs. 2, 6h). Periods of high δ^13^C between ~7.7 and ~5.6 ka and between ~4.5 and ~3.2 ka, indicative of strengthened NADW, correspond to the millennial-scale plateau and decrease in the ^231^Pa/^230^Th record, respectively (Fig. 6g, h). SCW thus apparently more than compensated strengthened NADW influence off South Africa, calling for a potentially major role of SCW in ventilating the Southern Ocean during these periods. Similarly as AAT, SST and SWW proxies, the ^231^Pa/^230^Th record correlates with projection ages significantly (n = 50, p < 0.01) within a millennial-scale time window that encompasses the ~500 yr transpacific water flow induced lag, highlighting a possible correspondence of precession-scale weakening of SCW in the Southern Ocean and ventilation in the NW Pacific during the Holocene. For the period after 8.1 ka, first difference correlations between the two records peak significantly (n = 50; p < 0.01) within the 500 ± 300 yr lag window (see Supplementary Material). Although to be regarded with care in view of low temporal resolution of the ^231^Pa/^230^Th record, inferences from AAT, SST and SWW data with regard to ocean overturning intensities and their possible relation to NW Pacific ventilation changes appear, at least for the period after 8.1 ka, supported by variations in SCW flow intensities.

Peaks of significant correlations of original (n = 50; p < 0.01) and first difference (n = 50; p < 0.05) data between the δ^13^C record and projection ages occur at projection age lags of ~400 yr (see Supplementary Material), which appear too short to warrant direct control of NADW on NW Pacific ventilation changes. NADW intensity changes could have nevertheless contributed to the dynamics of invigorated upwelling in the Southern Ocean and subsequently to the ventilation of the NW Pacific. As a further implication, if Southern Ocean overturning largely modulated mid-depth NW Pacific ventilation with a lag of roughly 500 yr, the emerging tendency of an in-phase relationship between NADW intensities^29^ and Southern Ocean overturning would argue against a scenario of a steady bipolar seesaw during the Holocene, which would be in contrast to the behaviour of the global ocean during the last glacial and deglacial period.

Although modulation of NW Pacific mid-depth waters in response to dynamical processes in the Southern Ocean seems a possible scenario, it still relies on mostly indirect inferences and the efficiency of physical processes such as wind-induced ocean overturning. Continuing research is therefore encouraged to evaluate the potential value of North Pacific margin sediments help tracing the Holocene history of the deep and intermediate Southern Ocean.

## Methods

### Lithology of PC C9002A

The sediments of the upper 9.5 m of PC C9002A, which comprise the last ~12 kyr, are dominantly composed of olive black to olive grey diatomaceous silty clay with occasional occurrences of sandy clay in silty clay. Ash layers are intercalated between 732.5 and 733 cm, 884 and 884.7 cm, and 893.3 and 894.3 cm (ref. 12).

### Age model for the Holocene section of PC C9002A

In order to construct an age model for PC C9002A and to reconstruct mid-depth water radiocarbon contents, ^14^C contents of foraminiferal shells were determined. Sediment samples were wet-sieved over a 63 μm sieve, rinsed in distilled water and dried overnight at 50°C. Using a binocular microscope, 1 to 9 mg of benthic and planktonic foraminifera were hand-picked from each sample and cleaned by soaking in a 30% H_2_O_2_ solution to remove adhering contaminants. Radiocarbon measurements were conducted at the AMS facility NIES-TERRA, Tsukuba, according to procedures described in Uchida et al.^62^,^63^.

Conventional ^14^C ages of planktonic foraminifera were recalculated to calendar ages based on the IntCal09/Marine09 calibration curve^25^ using the CALIB 6.0 software (http://calib.qub.ac.uk/calib/) (Table 1). While surface waters at site C9002 were dominantly influenced by the Oyashio current during the earliest Holocene, influence increasingly shifted to the Tsugaru current since ~10.6 ka^64^. We therefore applied a current-regime dependent reservoir age correction (ΔR) of 415 ± 105 yr before 10.6 ka, as based on Holocene ΔR values (n = 9) in the Oyashio current off the Kuril islands (CALIB Marine Reservoir Correction Database; http://calib.qub.ac.uk/marine/) and linearly interpolated ΔR values from 415 yr to 130 yr at 3.5 ka (Table 1). The latter value of 130 yr assumes mixing of 65% Tsugaru current water with a modern ΔR of 34 ± 42 yr in the eastern Tsugaru Strait^13^ and 35% Oyashio current water. This mixing ratio is based on observations of a 1:1 ratio between the two currents ~100 km south of site C9002A^14^, biased towards the Tsugaru current considering our core site's relative proximity to the Tsugaru Strait. We held ΔR uncertainties constant at ±105 yr throughout the time series to allow for larger ΔR variability.

In the Holocene section of PC C9002A calendar ages appear robust against variations in relative amounts of various species per sample based on independent preparations and measurements of two neighbouring samples of mixed planktonic foraminifera at 349.6 and 350.6 cm depth, which yielded an identical ^14^C age of 4346 yr (Table 1). The relatively high linear sedimentation rate (LSR), ~133 cm/kyr in the earliest Holocene between ~11.6 and ~10.3 ka and ~72 cm/kyr between ~10.3 and ~3.5 ka (Fig. 7), further minimizes sediment mixing influence on ^14^C dates. For the interval between core top and 249.5 cm (uppermost age control point) we assume a LSR of ~72 cm/kyr, which is defined by the condition that the core top was deposited at 0 ka.

### Benthic radiocarbon contents

**Figure 3:**
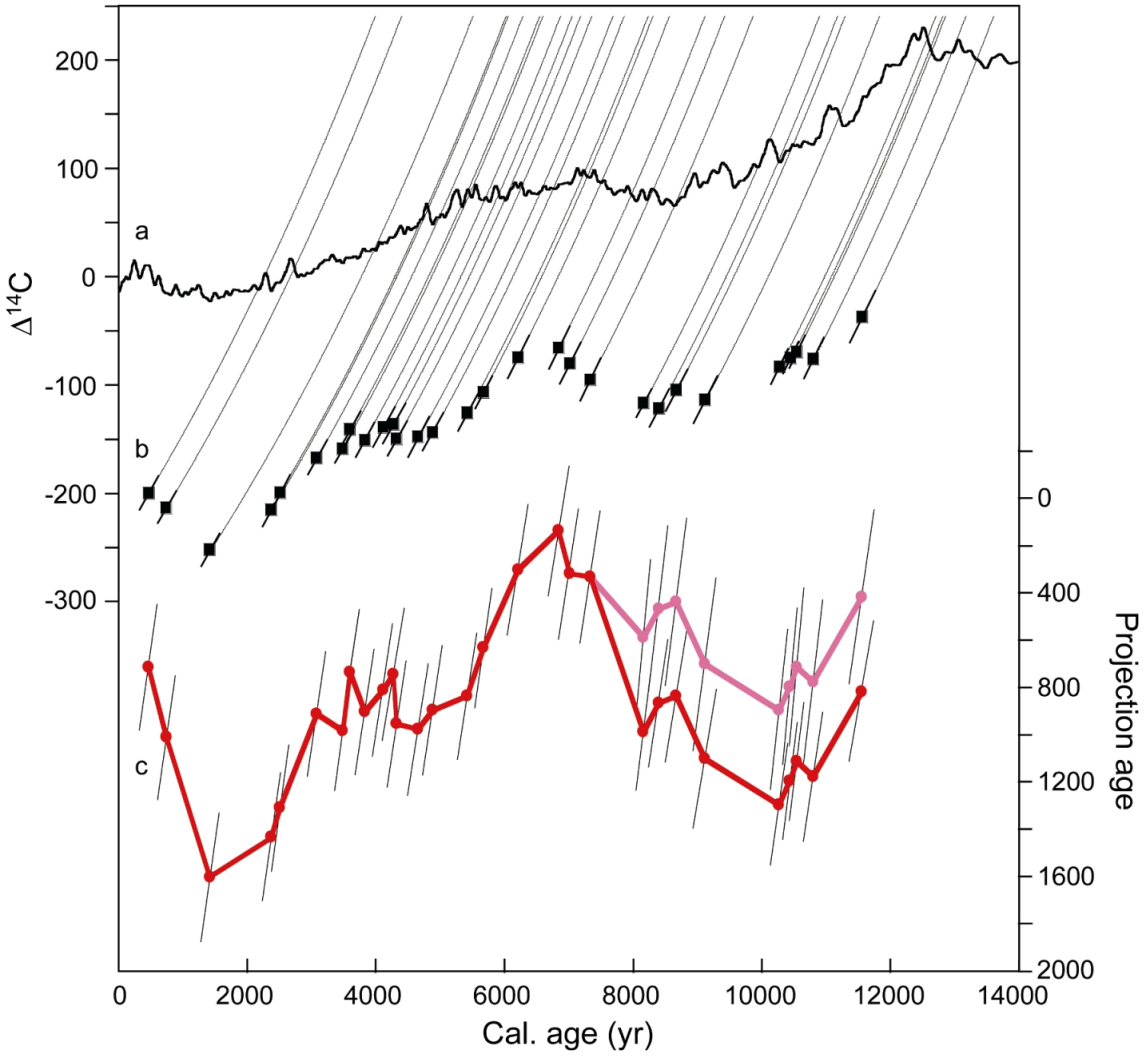
Composite of (a) atmospheric Δ^14^C (ref. 27), (b) mid-depth water Δ^14^C and (c) projection ages from PC C9002A off Shimokita peninsula. In (c) assumed source regions reservoir ages are ~1000 yr (red) and ~1400 yr (pink) (error bars as described in Methods; y-axis reversed). Closed-system ^14^C trajectories between mid-depth water and atmospheric Δ^14^C are indicated by thin lines.

**Figure 4:**
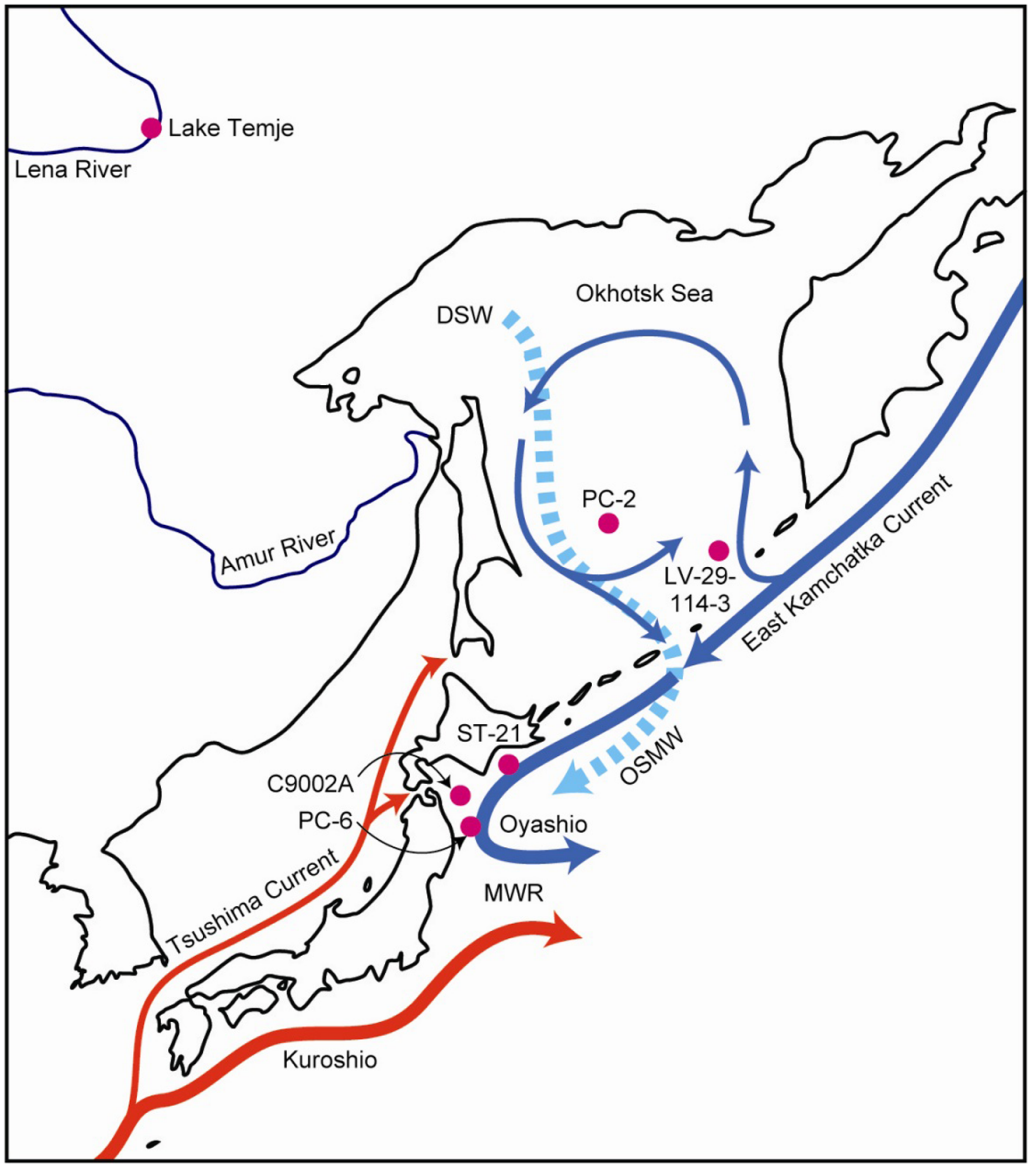
Map of the Northwest Pacific and adjacent land masses. Cold (blue arrows) and warm (red arrows) surface currents, the mixed water region (MWR) at the Kuroshio-Oyashio confluence and the Amur and Lena rivers are shown. The dashed blue arrow schematically illustrates sinking of dense shelf waters (DSW) and outflow of Okhotsk Sea Mode Water (OSMW) to the Northwest Pacific. Sediment core locations mentioned in this study are indicated as pink dots; from north to south the Lake Temje sediment core^38^, PC-2 (ref. 40), LV-29-114-3 (ref. 39), ST-21 (ref. 21), C9002A (this study) and PC-6 (ref. 45). Blank world map source: http://english.freemap.jp/world_e/8.html/.

**Figure 5:**
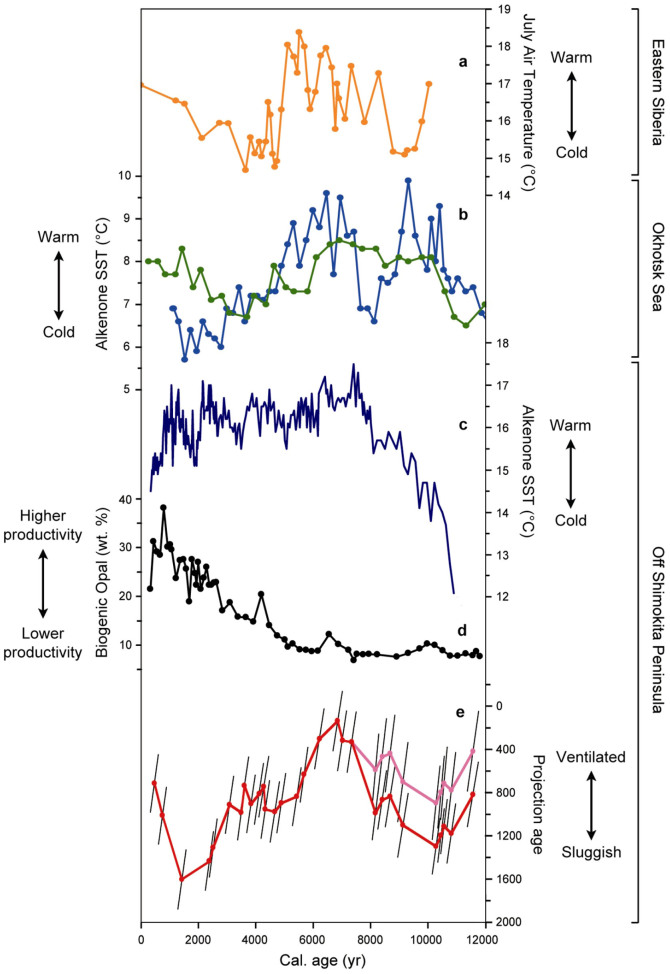
Comparison of variations in Northwest Pacific mid-depth water projection ages with atmospheric and oceanic records from Eastern Siberia, the Okhotsk Sea and the Northwest Pacific. (a) Chironomid-based July temperatures from Lake Temje^38^; (b) alkenone-based sea surface temperatures (SST) from the Okhotsk Sea cores PC-2 (green)^40^ and LV-29-114-3 (blue)^39^; (c) alkenone-based SST and (d) biogenic opal contents from core PC-6 off northeastern Honshu Island^45^; (e) projection ages for assumed source regions reservoir ages of ~1000 yr (red) and ~1400 yr (pink) (this study; error bars as described in Methods; y-axis reversed).

**Figure 6:**
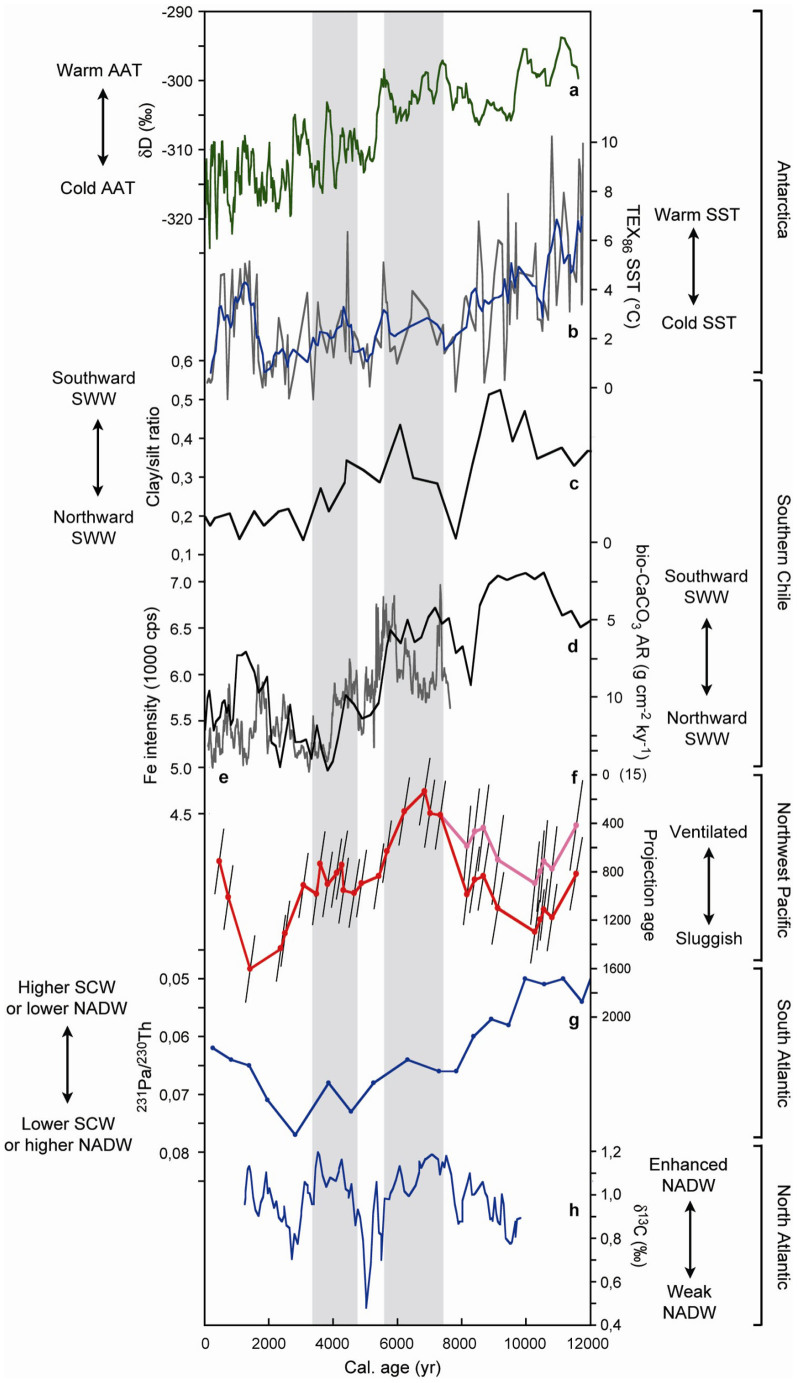
Comparison of variations in Northwest Pacific mid-depth water projection ages with atmospheric and oceanic records from the Southern Hemisphere and North Atlantic. (a) 5-point moving average (5-p.m.a.) of deuterium isotopes from Taylor dome as a proxy for Antarctic air temperature (AAT)^53^; (b) TEX_86_-derived sea surface temperatures (SST) (gray; blue: 5-p.m.a.) from off western Antarctic Peninsula (ODP site 1098)^49^; the Chilean data sets in panels c, d, and e are proxies for the latitudinal position of the southern westerly winds (SWW): (c) clay/silt ratios from the Seno Skyring fjord system in southern Chile (~53°S; core Sk1)^47^, (d) biogenic carbonate accumulation rates (bio-CaCO_3_ AR) from the fjord site Palm2 in southern Chile (~53°S; y-axis reversed)^47^, (e) 5-p.m.a. of Fe XRF-scanner intensity from the southern Chile continental slope (41°S; core GeoB 3313-1)^55^; (f) projection ages for assumed source regions reservoir ages of ~1000 yr (red) and ~1400 yr (pink) (this study; error bars as described in Methods; y-axis reversed); (g) ^231^Pa/^230^Th record from Cape Basin off South Africa (core MD02-2594; 2440 m water depth; y-axis reversed)^3^, SCW = Southern Component Water, NADW = North Atlantic Deep Water; (h) NADW record based on Δ^13^C of *Cibicidoides Wuellerstorfi* from the eastern North Atlantic (ODP site 980; 2179 m water depth)^30^. Gray shadings indicate periods of enhanced and plateauing ventilation off Shimokita peninsula during the mid- and early late Holocene.

**Figure 7:**
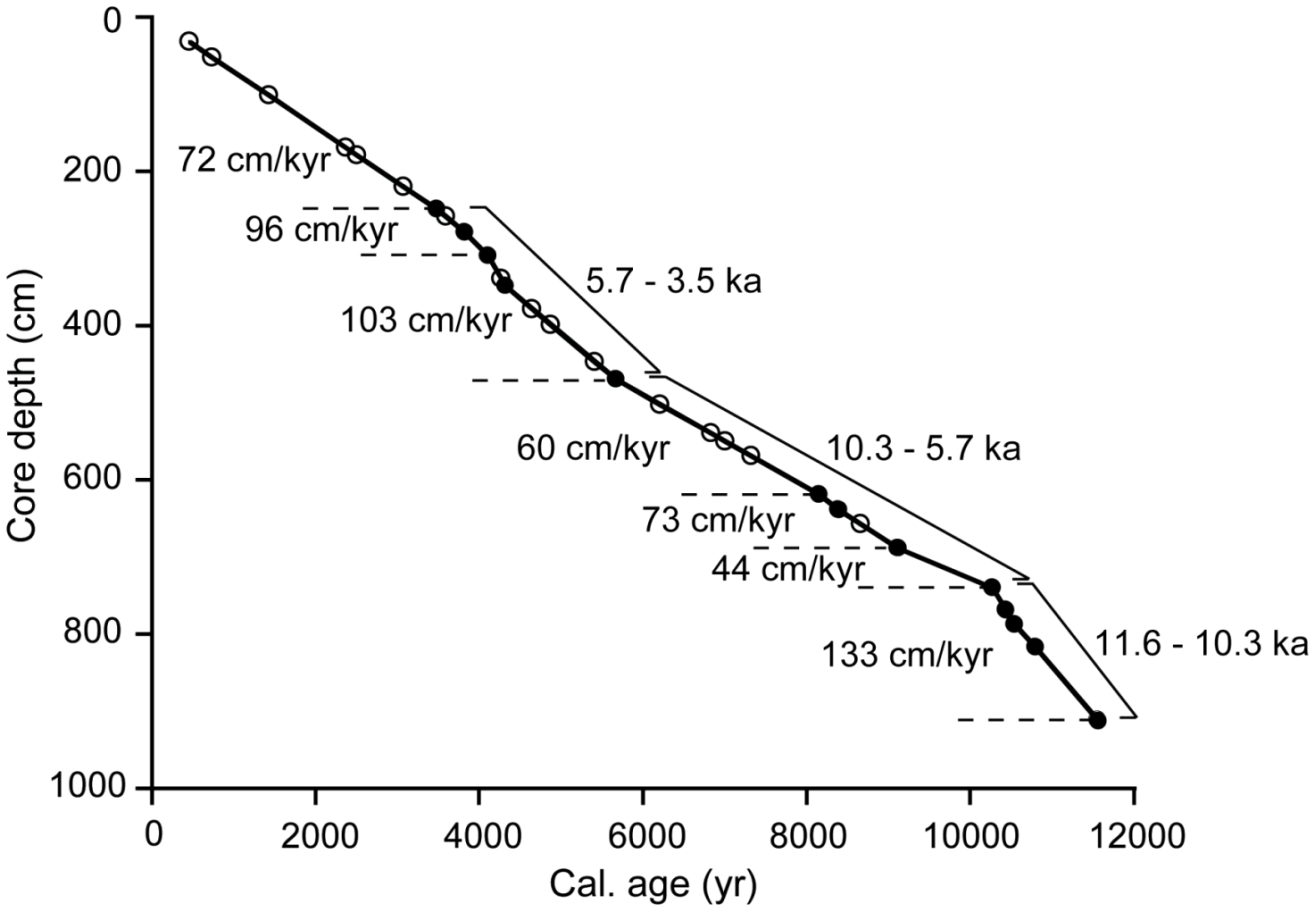
Age-depth diagram for the upper 9.2 m of PC C9002A. Solid circles denote measured calendar ages (age control points), open circles interpolated calendar ages. Sedimentation rates are indicated. Linear regression was performed through the age control points of each of the three time intervals indicated (11.6 to 10.3 ka, 10.3 to 5.7 ka, and 5.7 to 3.5 ka), in order to assess uncertainties in interpolated calendar ages due to possible variations in sedimentation rates between age control points (see Methods).

Age-corrected radiocarbon contents (Δ^14^C) of benthic foraminifera were calculated following the conventions of Stuiver and Polach^65^. For sediment layers, where planktonic ^14^C ages could not be determined due to insufficient amount of shells, we linearly interpolated between age control points to yield growth ages of the benthic foraminifera, i.e. the time at which the benthic foraminifer was alive (Fig. 7). Analytical uncertainties of calendar ages were similarly interpolated between age control points for an estimate of hypothetical analytical uncertainties of interpolated growth ages. Furthermore, in an effort to take into account possible LSR variations between age control points, which could affect interpolated benthic foraminiferal growth ages, we applied additional errors to interpolated calendar ages defined as the 1σ standard deviations (s.d.) of the distances between age control points and the respective regression line through age control points at three intervals in the core, between ~11.6 and ~10.3 ka, ~10.3 and ~5.7 ka, and ~5.7 and ~3.5 ka. These intervals were selected such as to reflect the typical variations in LSR over longer periods that are characterized by similar sedimentation rates (Fig. 7). The thus calculated additional uncertainties of interpolated calendar ages are 35 yr between ~11.6 and ~10.3 ka, 113 yr between ~10.3 and ~5.7 ka and 76 yr between ~5.7 and ~3.5 ka. In continuation, the latter value of 76 yr was also adopted for the period between ~3.5 and 0 ka. Uncertainties in benthic foraminiferal Δ^14^C combine estimated uncertainties in growth age of benthic foraminifera and analytical 1σ s.d. of benthic ^14^C ages (Fig. 3b; Table 1). Δ^14^C errors are dominated by calendar age uncertainties that in turn mainly depend on reservoir age uncertainties.

### Projection ages

To deduce ventilation ages, we employed the projection age method^27^, which takes into account that waters in the ocean interior commonly derive from distant regions, where they were influenced by the then current atmospheric Δ^14^C and reservoir age of the source regions. Following the method, we projected core site Δ^14^C values back along a closed-system ^14^C trajectory (i.e. assuming absence of mixing with younger or older water) to the intersection point with the IntCal09 atmospheric Δ^14^C curve (Fig. 3a, b). The difference in calendar ages of the intersection and core site yielded the projection age relative to the atmosphere. By subtracting the average reservoir age of primary source regions, we determined the projection age relative to the surface ocean in the source regions. We thereby assumed a constant source regions reservoir age of 1000 ± 215 yr, based on average reservoir ages retrieved from the CALIB Marine Reservoir Correction Database (http://calib.qub.ac.uk/marine/) for potential main source regions, 1100 ± 180 yr in the Southern Ocean south of 60°S (n = 16) and 900 ± 115 yr in the Okhotsk Sea north of 46°N (n = 5; points closest to the primary NPIW formation region). Since the reservoir ages of these source regions are similar and could be regarded as fairly constant during the Holocene^28^,^66^,^67^,^68^, the average value of 1000 ± 215 yr appears relatively robust. An exception may have constituted Southern Ocean reservoir ages of 1930 yr ± 290 (2σ) in the early Holocene^28^ that we considered as potential alternative scenario by assuming an average source regions reservoir age of 1400 ± 310 yr prior to 8.1 ka. Absolute variations in projection ages depend on the validity of core site and source areas reservoir age models and the assumed absence of ocean mixing effects involving different radiocarbon values along the flow path between the source regions and core site. Considering the smooth curvature in atmospheric Δ^14^C, relative changes in projection ages would also retain a similar expression, if assuming an approximately constant anomaly to closed-system ^14^C decay during the Holocene.

Uncertainties in projection ages (Figs. 2c, 3f) represent uncertainties in benthic Δ^14^C values projected to the atmospheric Δ^14^C curve and thus dominantly depend on reservoir age errors at the core site and source regions. In view of the relatively large variations in projection ages, departures from the proposed reservoir age model would not essentially change our results as long as reservoir age changes retain a smooth trend between neighbouring data points, which is plausible, since surface current intensities are not expected to vary abruptly during the Holocene.

## Author Contributions

M.U. managed the research work as member of the paleoceanographic research team during CK05-04 cruise. M.U. and S.F.R. designed the research and M.U. and S.F.R. wrote the manuscript. S.F.R. picked foraminifera and M.U. conducted radiocarbon analysis on foraminifera.

## Supplementary Material

Supplementary Information Supplementary Material

## Figures and Tables

**Figure 1:**
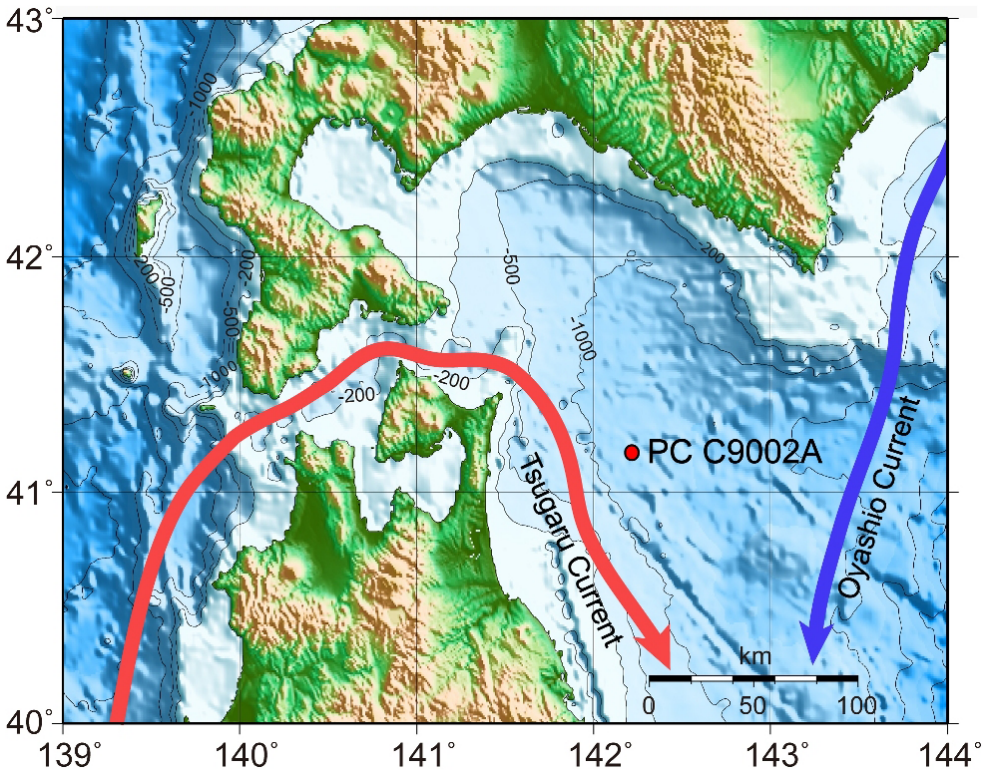
Map showing the location of piston core C9002A (red dot), bathymetry (in meters), the warm Tsugaru current (red arrow) and the cold Oyashio current (blue arrow) off Shimokita peninsula in northeastern Japan. Map created using the GMT software (http://gmt.soest.hawaii.edu/) and the ETOPO2v2 Global Gridded 2-minute Database (http://www.ngdc.noaa.gov/mgg/fliers/01mgg04.html/).

**Table 1:** Results on planktonic and benthic radiocarbon ages, calibrated calendar ages, age-corrected Δ^14^C, projection ages and their respective uncertainties (see Methods) in PC C9002A, and assumed reservoir age corrections for the area off Shimokita peninsula (see Methods). Prior to 8.1 ka, projection ages for both 1000 yr (left) and 1400 yr (right) source regions reservoir ages are indicated. Abbreviations: Cal. age = Calendar age; p.f. = planktonic foraminifera; b.f. = benthic foraminifera; N.p. = *Neogloboquadrina pachyderma*; G.b. = *Globigerina bulloides*; B.s. = *Bolivina spissa*; U.a. = *Uvigerina akitaensis*; E.b. = *Elphidium batialis*

Sample ID	Core depth (cm)	Species (p.f.)	Species (b.f.)	^14^C age (yr) (p.f.)		Reservoir age	Cal. Age (yr) (−1σ)	Cal. Age (yr)	Cal. Age (yr) (+1σ)	Cal. age (yr) (interpolated)	^14^C age (yr) (b.f.)		Δ^14^C (‰)		Projection age (yr)			
Value	(±1σ)	Value	(±1σ)	Value	±										Value		±	
1–34	33,2		Mixed							464	2299	59	−199,8	15,7	716		270	
1–54	53,1		Mixed							741	2704	38	−213,1	14,8	1014		266	
1–103	101,7		Mixed							1420	3698	88	−251,7	15,8	1600		277	
2–21	170,1		B.s., U.a.							2375	4310	72	−214,9	16,0	1435		273	
2–31	180,0		B.s., U.a.							2514	4286	71	−199,3	16,2	1311		273	
2–71, −72, −73	220,7		Mixed							3081	4519	47	−166,9	16,0	914		267	
2–101	249,5	Mixed	Mixed	3661	30	130	3361	3483	3621	3483	4828	34	−158,4	13,7	987		254	
2–111	259,4		U.a., E.b.							3597	4773	36	−140,8	17,0	738		270	
2–131	279,2	Mixed	Mixed	3955	36	143	3662	3825	3979	3825	5086	31	−150,5	16,6	905		269	
3–11	310,0	N.p., G.b.	E.b.	4181	30	153	3929	4113	4289	4113	5256	35	−138,9	19,1	812		283	
3–41, −42	340,2		U.a., B.s., E.b.							4275	5385	27	−135,8	19,1	745		282	
3–51	349,6	Mixed	U.a., B.s.	4346	38	161	4157	4325	4475	4325	5560	30	−149,3	16,6	955		269	
3–52	350,6	Mixed	Mixed	4346	65													
3–81, −82	379,4		Mixed							4656	5868	65	−147,9	19,0	979		283	
3–101, −102	399,7		Mixed							4882	6045	43	−143,3	18,1	898		277	
3–150	447,8		Mixed							5418	6401	30	−125,6	17,4	837		271	
4–21	470,6	N.p.	Mixed	5454	66	210	5523	5672	5800	5672	6474	30	−106,5	15,4	633		258	
4–53, −54	502,9		Mixed							6212	6713	40	−74,2	20,2	303		280	
4–91, −92	540,6		Mixed							6842	7251	35	−65,5	19,9	138		278	
4–102	551,0		Mixed							7016	7545	37	−79,9	19,6	319		278	
4–121	569,8		B.s., E.b., U.a.							7331	7983	70	−94,9	20,3	334		284	
5–21	619,4	N.p., G.b.	U.a., E.b.	7957	38	322	8025	8161	8268	8161	8984	31	−116,5	13,5	989	589	249	335
5–41, −42	639,8	Mixed		8204	38	333	8260	8394	8495	8394								
5–42	640,3		Mixed							8401	9259	41	−121,1	18,0	869	469	273	353
5–60, −61	658,6		B.s., E.b., U.a.							8671	9369	40	−104,3	19,6	839	439	281	359
5–91	688,9		Mixed							9118	9885	51	−113,4	22,0	1102	702	297	372
5–91, −92	689,4	Mixed		8809	45	360	8989	9125	9316	9125								
6–21	740,5	N.p., G.b.	Mixed	9771	34	403	10159	10273	10450	10273	10736	35	−82,9	16,6	1297	897	262	344
6–51	769,0	Mixed		9933	42	410	10317	10437	10552	10437								
6–52	769,9		Mixed							10442	10827	45	−74,5	14,7	1198	798	252	337
6–71	788,0	N.p.	Mixed	10046	38	415	10406	10545	10657	10545	10879	47	−69,0	15,2	1115	715	254	338
6–102	817,5	N.p.	Mixed	10234	45	415	10622	10802	10957	10802	11183	45	−75,2	19,5	1178	778	277	355
7–51	912,0		Mixed							11562	11592	46	−36,5	24,7	818	418	302	376
7–52	913,0	N.p.		10811	46	415	11316	11570	11724	11570								
