# Supplementary material for: A Southern Ocean trigger for Northwest Pacific ventilation during the Holocene?
Source: Sci Rep. 2014 Feb 17;4:4046. doi: 10.1038/srep04046 (PMC4027855; doi:10.1038/srep04046)
Supplement: Supplementary Information — Supplementary Material [file srep04046-s1.doc]

**Supplementary Material**

**Article Title: A Southern Ocean trigger for Northwest Pacific ventilation during the Holocene?**

Authors: S. F. Rella and M. Uchida

Contents:

1) Cross-correlations between projection ages and proxies from the Southern Hemisphere and North Atlantic

2) Figure S1: Results of cross-correlations between projection ages and proxies from the Southern Hemisphere and North Atlantic

3) Table S1: Summary of significant lags between projection ages and proxies from the Southern Hemisphere and North Atlantic

**1) Cross-correlations between projection ages and proxies from the Southern Hemisphere and North Atlantic**

Time series have been linearly interpolated between data points so that each time series could be correlated with projection ages at 50 equal distance data points that simulated the original variability structure sufficiently well. Cross-correlations between projection ages (assuming a Southern Ocean reservoir age of ~1900 yr prior to 8.1 ka) and the data sets from the Southern Hemisphere (SH) and the North Atlantic (NA) have been calculated for time lags between -2000 and 1000 yr, aiming to include the time scale of transpacific water mass transport and combined uncertainties in age models, whereby negative lag values indicate projection ages lagging SH and NA proxies (Fig. S1). Correlations and lags are discussed in their physically meaningful direction. For each lag, the calculations of mean and standard deviations used in cross-correlations were applied to the data points active in the correlation. Because the time series include more or less expressed trend components, the correlation between original data points emphasizes similarities in trends. In order to emphasize correlations in trend-corrected variabilities, correlations between first differences in projection ages and first differences in SH and NA proxies were calculated (Fig. S1). Correlations have been examined between the original data sets and additionally for the period after 8.1 ka to highlight correlations without the early Holocene, where specific environmental conditions in the aftermath of the late glacial period, such as meltwater influence, could have biased or overridden processes linking Antarctic temperature (AAT), sea surface temperatures (SST) off western Antarctic Peninsula and southern westerly winds (SWW) to ocean overturning. Results of correlations are shown together with lag-corrected 95% and 99% significance lines in Fig. S1. Lags that yield significant correlations with projection ages are summarized in Table S1.

**2) Figure S1: Results of cross-correlations between projection ages and proxies from the Southern Hemisphere and North Atlantic**


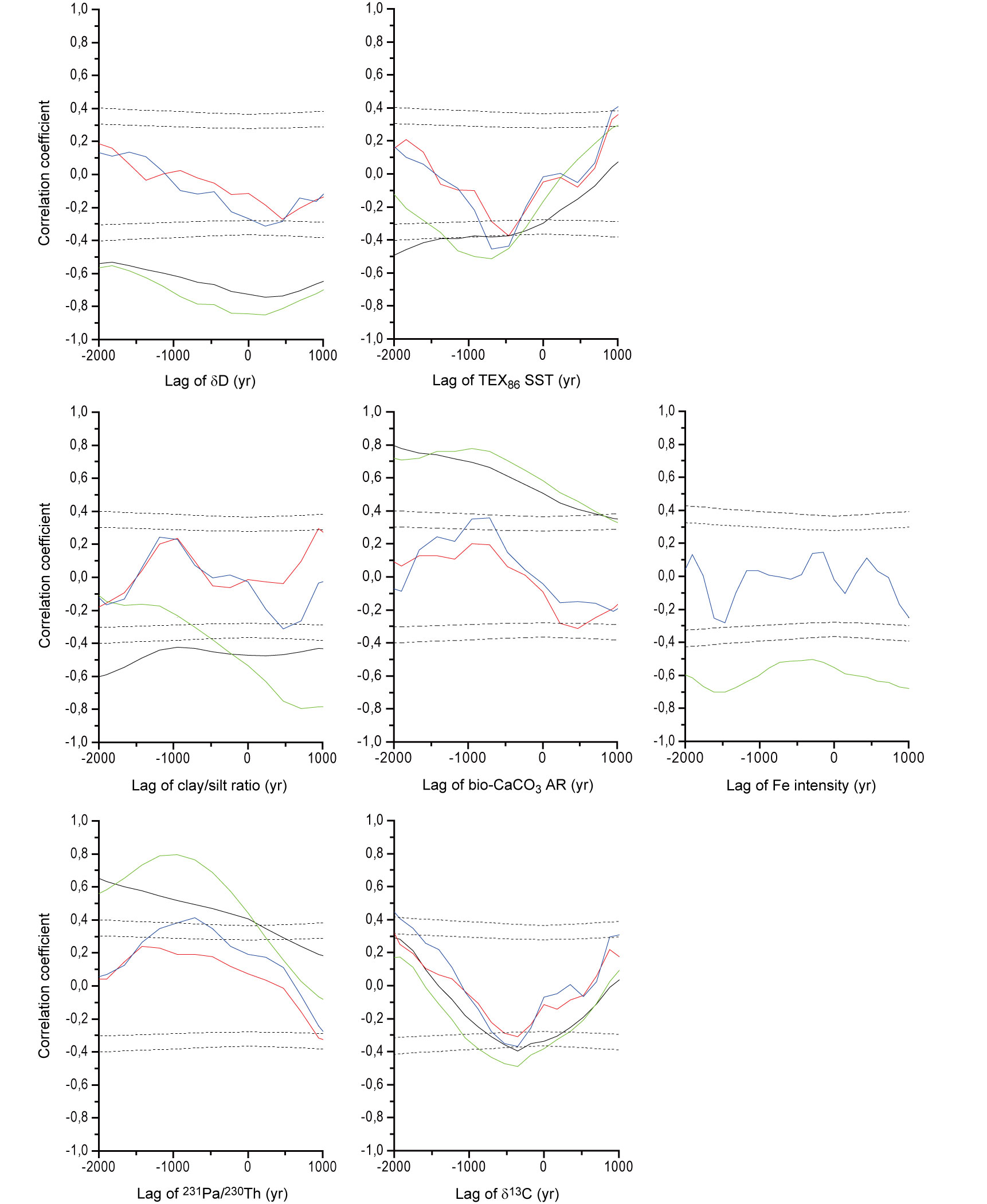


Figure S1: Results of cross-correlations between projection ages and proxies for AAT (δD), SST (TEX_86_), SWW (clay/silt ratio, Fe intensity, bio-CaCO_3_ accumulation rate (AR)), southern component waters (^231^Pa/^230^Th) and North Atlantic Deep Water (δ^13^C). Solid black and green lines indicate correlations between original data for the entire record and for the period after 8.1 ka, respectively. Solid red and blue lines indicate correlations between first differences for the entire record and for the period after 8.1 ka, respectively. Dashed lines delimit 95% and 99% significance levels.

**3) Table S1: Summary of significant lags between projection ages and proxies from the Southern Hemisphere and North Atlantic**

| Table S1: Summary of significant lags between projection ages and proxies from the Southern Hemisphere and North Atlantic examined between lags of -2000 and 1000 yr, wherein negative lags denote a lag of projection ages. Abbreviations: n.s. = not significant; Min = Minimal significant lag; Max = Maximal significant lag; SCW = southern component waters; NADW = North Atlantic Deep Water. | | | | | | | | |
| --- | --- | --- | --- | --- | --- | --- | --- | --- |
| Proxy | | δD (AAT) | TEX_86_ (SST) | clay/silt (SWW) | bio-CaCO_3_ AR (SWW) | Fe intensity (SWW) | ^231^Pa/^230^Th (SCW) | δ^13^C (NADW) |
| Significance | **Significant lags with projection ages (years)** | | | | | | | |
| p<0.01 | Min Max | -2000 1000 | -2000 -410 | -2000 1000 | -2000 720 |  | -2000 150 | -460 -240 |
| p<0.01 (<8.1 ka) | Min Max | -2000 1000 | -1260 -310 | -500 1000 | -2000 780 | -2000 1000 | -2000 120 | -870 40 |
|  | **Significant lags with projection ages in first differences (years)** | | | | | | | |
| p<0.01 | Min Max | n.s. | -470 -450 | n.s. | n.s. |  | n.s. | n.s. |
| p<0.01 (<8.1 ka) | Min Max | n.s. | -770 -390 | n.s. | n.s. | n.s. | -970 -570 | n.s. |
| p<0.05 | Min Max |  | -690  320 | n.s. | n.s. |  | n.s. | -530 -280 |
| p<0.05 (<8.1 ka) | Min Max | 70 450 | -850 -310 | 420 600 | -1060 -630 | n.s. | -1340 -320 | -680 -210 |
